# NOTCH1 Signalling: A key pathway for the development of high-risk chronic lymphocytic leukaemia

**DOI:** 10.3389/fonc.2022.1019730

**Published:** 2022-10-13

**Authors:** Jennifer Edelmann

**Affiliations:** Department of Hematology, Oncology, Palliative Care and Infectious Diseases, Alb Fils Kliniken, Göppingen, Germany

**Keywords:** CLL (chronic lymphocytic leukemia), NOTCH1, high-risk, review, mutation

## Abstract

NOTCH1 is a cell surface receptor that releases its intracellular domain as transcription factor upon activation. With the advent of next-generation sequencing, the *NOTCH1* gene was found recurrently mutated in chronic lymphocytic leukaemia (CLL). Here, virtually all *NOTCH1* mutations affect the protein’s PEST-domain and impair inactivation and degradation of the released transcription factor, thus increasing NOTCH1 signalling strength. Besides sequence alterations directly affecting the *NOTCH1* gene, multiple other genomic and non-genomic alterations have by now been identified in CLL cells that could promote an abnormally strong NOTCH1 signalling strength. This renders NOTCH1 one of the key signalling pathways in CLL pathophysiology. The frequency of genomic alterations affecting NOTCH1 signalling is rising over the CLL disease course culminating in the observation that besides *TP53* loss, 8q gain and *CDKN2A*/*B* loss, *NOTCH1* mutation is a hallmark genomic alteration associated with transformation of CLL into an aggressive lymphoma (Richter transformation). Both findings associate de-regulated NOTCH1 signalling with the development of high-risk CLL. This narrative review provides data on the role of *NOTCH1* mutation for CLL development and progression, discusses the impact of *NOTCH1* mutation on treatment response, gives insight into potential modes of NOTCH1 pathway activation and regulation, summarises alterations that have been discussed to contribute to a de-regulation of NOTCH1 signalling in CLL cells and provides a perspective on how to assess NOTCH1 signalling in CLL samples.

## Introduction

The Notch family consists of four protein paralogs (NOTCH1-4) that are single-pass transmembrane receptors involved in cell fate decisions and cell differentiation by releasing a transcription factor upon receptor activation (1). De-regulated NOTCH signalling is frequently associated with malignant transformation of haematologic and solid cancers (2). Gain-of-function mutations of the *NOTCH1* paralog were first discovered in T-cell acute lymphoblastic leukaemia (T-ALL) with a frequency of 56% in a cohort of 96 samples taken at diagnosis (3). With regards to other haematologic malignancies, de-regulated NOTCH signalling was also discovered in B-cell lymphomas *via* mutations in the *NOTCH1* and *NOTCH2* genes. In chronic lymphocytic leukaemia (CLL), recurrent *NOTCH1* mutations were observed at all disease stages, whereas mutations in the paralogs *NOTCH2*, *NOTCH3* and *NOTCH4* were rare events with frequencies <1% (*NOTCH2* in 0.9%; *NOTCH3* in 0.7%; *NOTCH4* in 0.6%) (4).

In B-cell lymphoma, *NOTCH1* mutations are almost always located in exon 34 and affect the protein’s PEST-domain responsible for inactivation and degradation of the NOTCH1 intracellular domain (NICD1), which is released as transcription factor after NOTCH1 activation (5). This is different to findings made in T-ALL, where mutations are often located in the NOTCH1 heterodimerization domain (HD-domain). While PEST-domain *NOTCH1* mutations prolong transcription factor activity, mutations in the HD-domain disrupt the receptor’s autoinhibitory conformation and lead to a stronger dysregulation of signalling strength than PEST-domain mutations (increase by factor 1.5 to 2 for PEST-domain mutations, by factor 3 to 9 for HD-domain mutations and by factor 20 to 40 when both mutation types affect the same NOTCH1 allele) (3).

In contrast to HD-domain mutations, PEST-domain mutations can only exert pathogenic effects after NOTCH1 activation and NICD1 release. This at least partly depends on ligand-binding, which inflicts shear forces opening the receptor’s autoinhibitory domain and making a cleavage site accessible for the metalloenzymes ADAM10 and ADAM17. ADAM-mediated cleavage generates an intermediate cleavage-product termed NEXT (NOTCH1 extracellular truncation), which is ultimately cleaved by the gamma-secretase complex releasing NICD1 (6–8).

**Figure 1:**
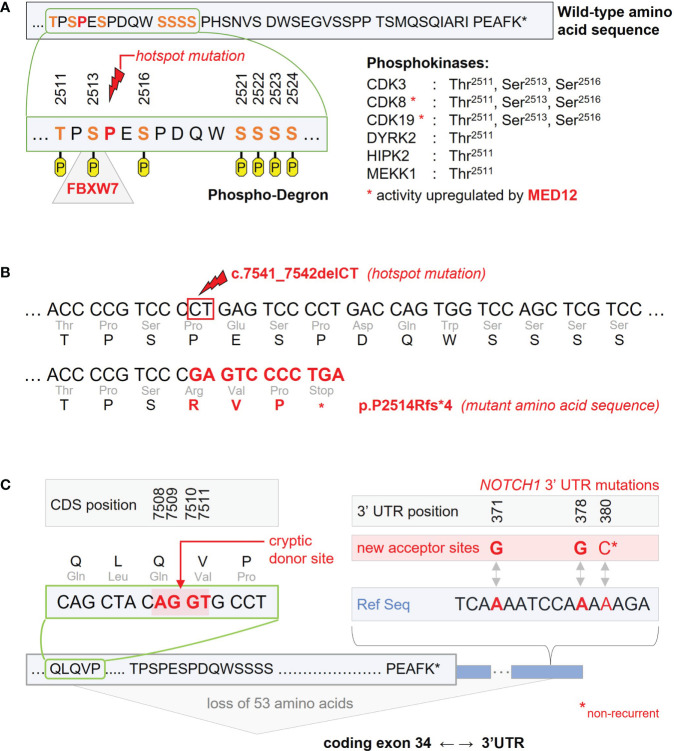
Recurrent *NOTCH1* gene mutations impairing inactivation and degradation of the NICD1 transcription factor. **(A)** C-terminal wild-type amino acid sequence encoded by *NOTCH1* exon 34. The amino acid sequence shown is part of the protein’s PEST-domain and held responsible for inactivation and ubiquitination of the NICD1 transcription factor released after NOTCH1 receptor activation. According to PhosphoSitePlus, the Thr^2511^, Ser^2513^, Ser^2516^, Ser^2521^, Ser^2522^, Ser^2523^, and Ser^2524^ amino acid residues were identified as phosporylation sites potentially involved in NICD1 inactivation and degradation. As part of this putative phospho-degron, Ser^2513^ has been identified as binding site for the ubiquitin ligase FBXW7 recurrently affected by inactivating mutations in CLL. The FBXW7 binding site is directly adjacent to the Pro^2514^ codon harbouring the c.7541_7542delCT hotspot mutation. Phosphokinases associated with Thr^2511^, Ser^2513^, and Ser^2516^ phosphorylation are CDK3, CDk8 and CDK19. Activation of CDK8 and CDK19 is at least partly mediated by MED12 found to be recurrently mutated in CLL. **(B)** C-terminal nucleotide and amino acid sequences as found with the c.7541_7542delCT hotspot mutation. The resulting frameshift mutation leads to a premature stop in the fourth altered codon. **(C)** Non-coding mutations in the 3’ untranslated region (3’ UTR) of *NOTCH1* induce a new acceptor site for alternative splicing. Recurrent single-nucleotide variants were found in position 371 (corresponding to chr9 position 139390152 in the GRCh37/hp19 reference genome) and in position 378 of the *NOTCH1* 3’UTR (chr9 position 139390145). A non-recurrent single-nucleotide variant was described for position 380 (chr9 position 139390143). Interaction with a cryptic donor site located in the coding part of exon 34 (positions 7508-7511 of the coding sequence, CDS) leads to loss of the 53 terminal amino acids.

Within the PEST-domain, a hotspot mutation could be identified accounting for >90% of *NOTCH1* mutant CLL cases. It represents a deletion of two nucleotides in the 2514 proline codon leading to a premature stop-codon in the fourth altered codon (c.7541_7542delCT, p.P2514Rfs*4). The other exon 34 mutations represent more proximal stop codons so that loss of the C-terminal amino acid sequence is a common characteristic of all PEST-domain mutations (at least 39 amino acids plus sequence alteration of the 3 preceding amino acids, **Figures 1A, B**) (5, 9).

## NOTCH1 mutation frequency in CLL

Screening CLL samples for *NOTCH1* mutations within prospective clinical trial cohorts by exon 34 targeted next-generation sequencing revealed an enrichment for *NOTCH1* mutations over the disease course. In monoclonal B-cell lymphocytosis (MBL, defined by <5000 CLL-phenotypic cells per µl peripheral blood) (10), *NOTCH1* mutations were found in 11% of cases compared to a frequency of 13% observed in early stage CLL (Binet A) not requiring treatment (frequencies in MBL and early stage CLL did not significantly differ when assessed within the same clinical trial, NCT00917450; p=0.6046 as inferred by Fisher’s exact test) (10). In unselected CLL patients needing first-line treatment, frequencies ranged from 17% to 23% (11, 12), in relapsed/refractory (R/R) CLL patients from 24% to 29% (13, 14). Sequencing studies outside of clinical trial cohorts and/or using less sensitive sequencing techniques revealed somewhat lower frequencies (9, 15–22). In Richter transformation (RT) comprising progression of CLL into aggressive lymphoma, *NOTCH1* mutation was identified as hallmark genomic alteration next to *TP53* alteration, *CDKN2A*/*B* loss and *MYC* gain. *NOTCH1* mutation frequencies were 25 and 41% in a limited number of RT-cases screened (N=28 and 27) (23, 24).

In addition to coding *NOTCH1* mutations, non-coding mutations were found in the 3’ untranslated region (UTR) with recurrent 139390152A>G and 139390145A>G sequence alterations (referring to the GRCh37/hg19 reference genome) (25, 26). Within the UK LRF CLL4 trial, non-coding *NOTCH1* mutations were identified in 2.4%, mutually exclusive from coding *NOTCH1* mutations found in 10.1% of patients. Both *NOTCH1* mutant patient groups had a comparable clinical outcome with inferior progression-free survival (PFS) as compared to patients with wild-type *NOTCH1*. This is in line with the biologic effect of 3’-UTR mutations leading to the loss of at least 53 terminal amino acids by creating a new acceptor site in the 3’-UTR and involving a cryptic donor site in the coding region of exon 34 or less frequently, the canonical donor site on exon 33 for aberrant splicing (**Figure 1C**) (25, 26).

## Impact of NOTCH1 mutation on the CLL disease course

Whether *NOTCH1* mutation initiates CLL development, was addressed in a study analysing multipotent hematopoietic progenitor cells flow-sorted from the bone marrow of CLL patients for sequence variations (27–29). Accounting for sorting impurities and demonstrating multipotency of progenitor cells by enforcing myeloid colony formation, *NOTCH1* mutations were identified in progenitor cells at unexpectedly high frequencies. The same accounted for other lymphoid oncogenes such as *BRAF*, *SF3B1*, *NFKBIE* and *EGR2* (27). In line with *NOTCH1* mutation being an early event in CLL development, functional analyses using a constitutively active form of NOTCH1 induced CLL disease onset in an IgH.TEµ mouse model and had an impact on direct and indirect cell-cycle regulation increasing the *in-vivo* proliferation rate of lymphoid cells (30). Moreover, the NOTCH1 target gene repertoire is supposed to initiate a broad program aiming at survival and proliferation of mature B-cells by including *MYC* and other genes involved in B-cell receptor and cytokine signalling (31).

Although the results outlined above strongly support a role for *NOTCH1* mutation as driver of CLL initiation and progression, this notion was not fully backed up when analysing sequential CLL samples for dynamics in *NOTCH1* mutant cancer cell fractions (CCFs). While some studies demonstrated an increase of *NOTCH1* mutant CCFs over the disease course or a constantly high mutation burden (4, 32), other studies identified individual cases with receding or disappearing *NOTCH1* mutant clones (33). Given the limited number of sequential samples analysed for the dynamics of *NOTCH1* mutant CCFs, more work needs to be invested to fully understand the behaviour of *NOTCH1* mutant CLL cells during periods of “watch & wait” and under the selective pressure of therapy.

## Impact of NOTCH1 mutation on response to treatment

The role of *NOTCH1* mutations in conferring treatment resistance was first assessed in the setting of chemotherapy. Analyses within the UK LRF CLL4 trial comparing chlorambucil mono versus fludarabine mono versus fludarabine plus cyclophosphamide (FC) identified *NOTCH1* mutation as an independent risk factor for shorter PFS and overall survival (OS) (26, 34). However, the unfavourable impact of *NOTCH1* mutation on response to chemotherapy was not reproducible in the FC-arm of the CLL8 trial and in the chlorambucil-arm of the CLL11 trial of the German CLL Study Group (GCLLSG) (9, 35).

In treatment arms combining an anti-CD20 monoclonal antibody (mAb) with chemotherapy, presence of *NOTCH1* mutation was associated with inferior PFS. This included the FC-rituximab-arm of the GCLLSG CLL8 trial, the ofatumumab-chlorambucil-arm in the COMPLEMENT-1 trial and the obinutuzumab-chlorambucil arm in the GCLLSG CLL14 trial (9, 11, 12). With regards to type I anti-CD20 mAbs (rituximab and ofatumumab), *NOTCH1* mutation could be identified as predictive marker for no or only weak benefit from anti-CD20 mAb addition to chemotherapy (9, 11, 17), whereas results obtained in the GCLLSG CLL11 trial implied that the type II anti-CD20 mAb obinutuzumab was able to overcome this non-benefit (35).

**Figure 2:**
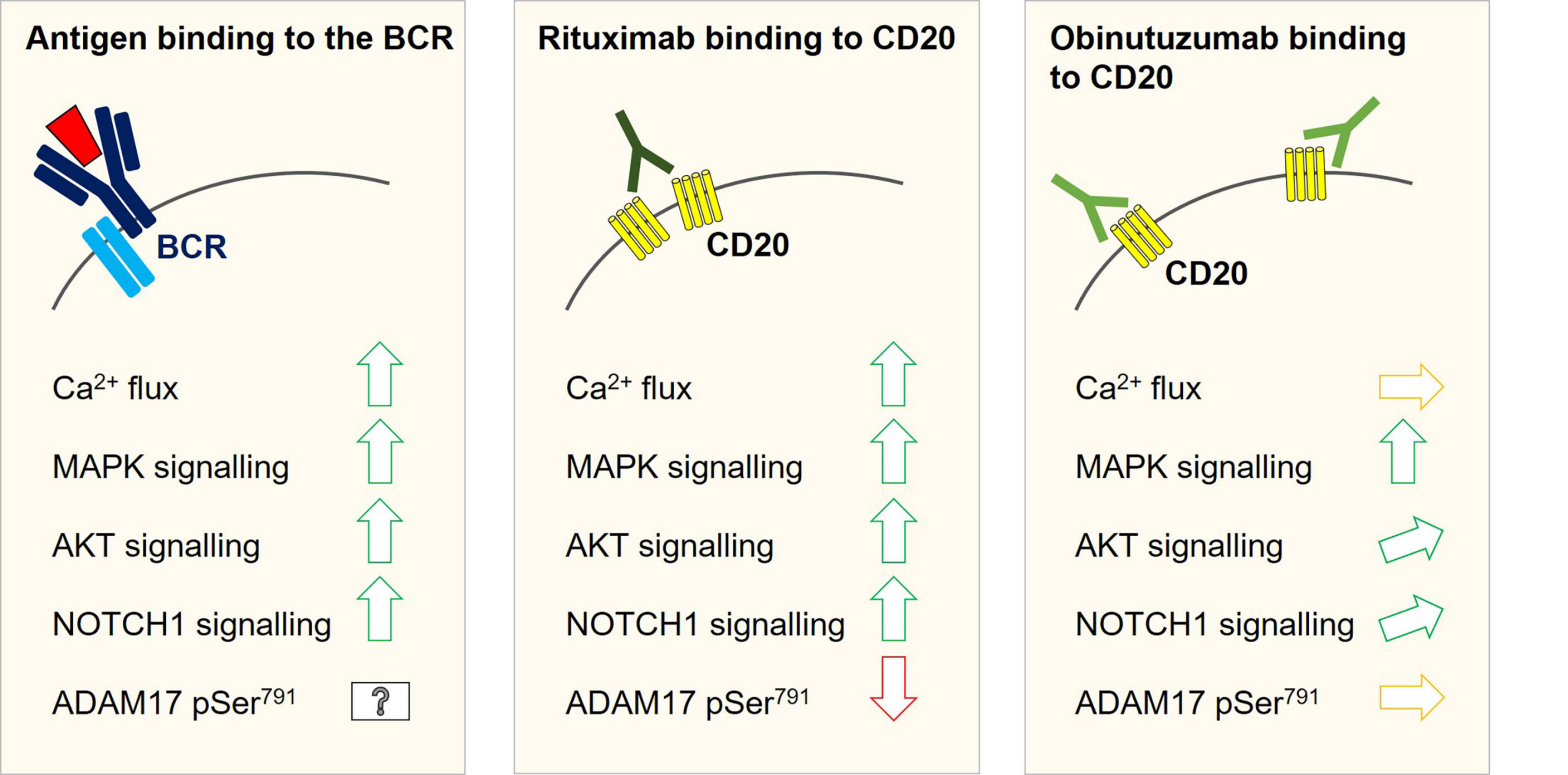
Intracellular signalling changes with a potential relevance for NOTCH1 activation as observed in SU-DHL4 cells after rituximab and obinutuzumab *in-vitro* treatment. Antigen binding to the B-cell receptor induces Ca^2+^-flux and activates the MAPK and Pi3K/AKT signalling pathways. All three signalling events have been linked to modulation of ADAM10/ADAM17 activity. Both metalloproteases were shown to promote the first cleavage step to release the NICD1 transcription factor. Likewise, an increase in NOTCH1 signalling could be associated with an activation of the B-cell receptor signalling cascade. As well as B-cell receptor activation, rituximab binding to CD20 can induce Ca^2+^-flux, MAKP signalling and AKT activation. In keeping with this notion, NOTCH1 signalling was shown to be inducible by rituximab treatment in SU-DHL4 cells. Phosphoproteomic studies in SU-DHL4 cells revealed a significant decrease in ADAM17 Ser^791^ phosphorylation at one hour after start of rituximab treatment. ADAM17 Ser^791^ de-phosphorylation has been associated with an increase in ADAM17 activity. Obinutuzumab binding to CD20 induces MAPK signalling, but only weak AKT activation and no measurable Ca^2+^-flux in SU-DHL4 cells. NOTCH1 signalling was induced to a much lower degree by obinutuzumab than by rituximab treatment and ADAM17 Ser791 phosphorylation was not significantly altered.

Regarding explanations for reduced benefit from rituximab and ofatumumab, studies demonstrated lower CD20 expression on the surface of *NOTCH1* mutant CLL cells reducing the antibodies’ capacity to elicit complement-dependent cytotoxicity (36, 37). This was explained by higher levels of active histone deacetylases (HDACs) in the nucleus of *NOTCH1* mutant cells due to greater disruption of RBPJ/HDAC protein complexes by increased nuclear protein levels of mutant NICD1 (36). Free HDAC1 and HDAC2 were shown to interact with the promoter of the CD20 coding gene *MS4A1* and to suppress its transcription (36, 38). However, this explanation remained contradictory since associations between low CD20 expression and *NOTCH1* mutation were not reproducible in the GCLLSG CLL8 nor in the COMPLEMENT-1 trial (9, 11). As another explanation, *in-vitro* studies on SU-DHL4 revealed strong activation of NOTCH1 signalling by rituximab, but not obinutuzumab, possibly explained by distinct intracellular signalling events that both anti-CD20 mAbs induce in the B-cell receptor (BCR) signalling cascade (**Figure 2**) (39). Exceedingly strong transcriptional changes induced after release of mutant NICD1 in concert with pro-survival signalling changes following rituximab binding to CD20 could thus also be responsible for a higher resistance of *NOTCH1* mutant CLL cells towards rituximab-based chemo-immunotherapy. Taken together, the reasons for reduced benefit of NOTCH1 mutant CLL from type I anti-CD20 mAbs are not sufficiently understood and warrant further research.

Modulation of NOTCH1 signalling through BCR signalling, as suggested above, was supported by *in-vitro* and *in-vivo* data obtained under BTK inhibition, since ibrutinib treatment was shown to suppress NOTCH1 signalling (39, 40). In keeping with this notion, the presence of *NOTCH1* mutation had no negative impact on response to ibrutinib treatment in the RESONATE trial (13). Suppressive effects on NOTCH1 signalling were also found for the Pi3K-inhibitor idelalisib in an *in-vitro* setting (39), whereas clinical data based on nine patients suggested poor response of *NOTCH1* mutant CLL patients to idelalisib (41).

With regards to the BCL2-inhibitor venetoclax, a pooled dataset on results from monotherapy showed that *NOTCH1* mutation was associated with a shorter duration, but not probability of response (40). In the MURANO trial combining venetoclax with rituximab, *NOTCH1* mutation had no adverse impact on PFS, but was associated with lower rates of undetectable minimal residual disease (MRD) at the end of treatment (20). The GCLLSG CLL14 trial combining venetoclax with obinutuzumab did not reveal a negative impact of *NOTCH1* mutation on PFS and MRD rates (12).

## Activation of the NOTCH1 receptor

In the lymph node, CLL cells were shown to frequently express high NICD1 protein levels. Hence, presence of the NOTCH1 ligands JAG1, JAG2, DLL1, DLL3, and DLL4 expressed by microenvironmental cells in the lymph node constitutes one regulative factor for NOTCH1 activation (42, 43). In the perinodal area, CLL cells were shown to express only low levels of the NICD1 transcription factor suggesting that NOTCH1 signalling rapidly decreases once cells exit their lymph node niche (42, 43). This is in line with the relatively short half-life of few hours described for the NICD1, which allows a dynamic regulation of NOTCH1 target genes (44–46). In contrast to these findings, about 50% of CLL cases lacking a *NOTCH1* mutation present with high NOTCH1 signalling levels in virtually all peripheral CLL cells suggesting a continuous induction of NOTCH1 cleavage in the blood stream (31). This may occur by ligand-dependent mechanisms such as interaction with other CLL cells expressing JAG1 and JAG2 or interaction with ligand-expressing endothelial cells (31).

Alternatively, a ligand-independent mode of NOTCH1 activation could be responsible for continuously high signalling levels. This mode is less well understood, likely executed by ADAM17 and possibly occurring in the intracellular compartment where NOTCH1 is expressed on endosomal membranes (47, 48). Importantly, ligand-independent NOTCH1 cleavage was described to occur shortly after activation of T-cell as well as B-cell receptors (39, 49–54), a finding that associates NOTCH1 signalling with the response of T- and B-cells to antigen recognition and is interesting against the notion that high frequencies of *NOTCH1* mutation could be associated with aggressive stereotyped B-cell receptor subsets (subsets #1, #6, #8, and #59) (55).

These notions raise questions on how ligand-independent NOTCH1 cleavage could be regulated. Liquid chromatography-tandem mass spectrometry conducted on samples from SU-DHL4 cells treated with rituximab for one hour revealed significant de-phosphorylation of ADAM17 Ser^791^ as compared to untreated and obinutuzumab treated cells, which coincided with an increase in NOTCH1 signalling (39). Metalloprotease activity is modulated by phosphorylation changes on the ADAM intracellular domain and ADAM17 Ser^791^ de-phosphorylation has been associated with an increase in ADAM17 activity (56). MAPK and Pi3K/AKT signalling were shown to be up-stream of ADAM10/ADAM17 phosphorylation changes (56–58). This potentially links antigen-induced BCR signalling to NOTCH1 activation. *In-vitro* results from SU-DHL4 cells revealed that MAPK and AKT activation can also be observed after rituximab treatment, whereas obinutuzumab is a strong activator of the MAPK pathway, but only a weak activator of AKT and NOTCH1 signalling (39). MAPK and particularly Pi3K/AKT may hence constitute links between the BCR signalling pathway and NOTCH1 cleavage (**Figure 2**). Against this background, it is interesting that a more recent study revealed coincidence of AKT overactivation with increased NOTCH1 signalling levels in an Eµ-TCL1 CLL mouse model (59). Taken together, these findings warrant a better understanding of the regulatory processes behind NOTCH1 cleavage to fully understand the NOTCH1 activation process in CLL cells.

## Regulation of NOTCH1 transcription factor activity

Upon translocation of NICD1 into the nucleus, the transcription factor gets integrated into a protein complex, termed coactivator complex. This complex encompasses the DNA-adapter protein RBPJ and chromatin modifiers to regulate expression of NOTCH1 target genes. RBPJ has a dual role in the transcriptional regulation of NOTCH1 target genes, since in the absence of NICD1 in the nucleus, it is integrated into a protein complex repressing NOTCH1 target gene transcription. Upon arrival of NICD1 in the nucleus, this repressor complex is disrupted allowing the formation of the coactivator complex formed around RBPJ (60).

Besides RBPJ, the NOTCH1 repressor complex consists of SPEN, CTBP, NCOR, CIR, possibly SNW1 and a histone deacetylase (60). Of note, *SPEN* was found recurrently mutated in CLL with a rising frequency over the disease course. An unselected CLL cohort needing first-line treatment revealed a *SPEN* mutation frequency of 1.8% (NCT00281918; 5/278 cases), a R/R-cohort a frequency of 3.7% (NCT01392079; 4/108 cases) and an RT-cohort a frequency of 18.5% (5/27 cases) (4, 14, 23). While in the first cohort, *SPEN* mutation was not found to co-occur with *NOTCH1* mutation, this was frequently observed at more advanced disease stages (1/4 cases in the R/R-cohort, 3/5 cases in the RT-cohort), suggesting synergistic effects of both mutations (4, 14, 23). Expression of two well-established NOTCH1 target genes (*HES1* and *DTX1*) was significantly increased in *SPEN* mutant primary CLL samples, possibly explained by de-repression of NOTCH1 target genes upon disruption of the NOTCH1 repressor complex (14).

With regards to other components of the corepressor complex, the *RBPJ* and *SNW1* gene loci were found to be recurrently deleted in high-risk CLL (minimally deleted regions: del (4)(p15.1-p15.2) for *RBPJ* loss; del (14)(q24.3-q32.1) for *SNW1* loss). *RBPJ* deleted CLL samples presented with higher *DTX1* but not *HES1* expression levels rendering del (4)(p15.2) an alteration, which may be linked to de-regulated NOTCH1 signalling (14). *SNW1* deleted CLL samples revealed no evidence for increased NOTCH1 target gene transcription (14).

As a well-established NOTCH1 target gene, *MYC* is recurrently affected by chromosomal gains (14, 61, 62). Large 8q-gains encompassing the *MYC* gene locus were found with a rising frequency over the disease course (~16% in high-risk cases) next to focal gains inside a *MYC* enhancer region (14, 31, 62). The latter contained NICD1 binding sites and represented the only recurrent focal gain found in CLL (frequency ~1% in unselected CLL cases) (62). As to what extent NOTCH1 signalling drives CLL progression *via* modulation of *MYC* transcription is yet unclear.

Notably, *SF3B1* mutation as one of the most frequently altered gene in CLL could also be associated with increased NOTCH1 signalling (63). This is particularly interesting against the notion that *NOTCH1* and *SF3B1* mutations virtually show mutual exclusivity (9). The *SF3B1* gene encodes a spliceosome component and its mutation was found to induce alternative splicing of the *DVL2* gene (63). DVL2 is described as negative regulator for transcription of NOTCH1 target genes *via* binding to RPBJ, whereas the identified *DVL2* splice variant is associated with up-regulated transcription of the NOTCH1 target gene *HES1* (63, 64). If this observation is explainable *via* RBPJ’s role in the NOTCH1 corepressor or activator complex, remains a subject for future investigation.

## Inactivation and degradation of the NOTCH1 transcription factor

The PEST-domain is responsible for inactivation and degradation of NICD1. The inactivation process is thought to involve phosphorylation of serine residues located inside and directly adjacent to the PEST-domain fragment that gets lost by the pP2514Rfs*4 mutation (65). CDK8 and its paralog, CDK19, are two kinases that have been associated with inactivating phosphorylation of NICD1 phosphorylation sites (65, 66). In addition, the NOTCH1 Ser^2513^ residue was identified as binding site for the FBXW7 ubiquitin ligase targeting NICD1 for proteasomal degradation (**Figure 1A**) (67). Interaction between NICD1 and FBXW7 was particularly shown for the α- (nucleoplasmic) and γ-isoform (nucleolar) of FBXW7, but not for its β-isoform (cytoplasmic), suggesting that NICD1 interacts with FBXW7 inside the nucleus (67, 68).

Mutations in the *FBXW7* gene were found in 4% of previously untreated CLL patients (36/905). They frequently affect hotspots in the protein substrate binding domain leading to a reduced binding capacity of FBXW7 to NICD1 and other proteins. NICD1 protein levels were increased in *FBXW7*-mutant CLL cases, comparable to findings made in *NOTCH1*-mutant CLL cases (68). However, if accumulating non-ubiquitinated NICD1 retains transcription factor activity or is at least partly inactivated by phosphorylation or other signalling events needs further investigation.

Another genomic event possibly involved in NOTCH1 de-regulation is *MED12* mutation. In a meta-analysis including 1429 samples from 5 studies, these mutations were discovered in 2.9% of CLL patients. Of note, they were found to be mutually exclusive from *NOTCH1* mutation. Similar to results obtained for *FBXW7* mutation, *MED12* mutations could be associated with increased NICD1 protein amounts (69). One possible explanation for NICD1 accumulation is that MED12 has been associated with CDK8 and CDK19 kinase activation (70–72).

## Discussion

The findings outlined above demonstrate that NOTCH1 de-regulation can occur at the level of NOTCH1 receptor activation, NOTCH1 target gene expression, and NICD1 inactivation. When assessing the impact of abnormally strong NOTCH1 signalling, it is hence not sufficient to only screen for *NOTCH1* gene mutations. Moreover, non-mutational NOTCH1 activation found in ~50% of CLL cells lacking a *NOTCH1* mutation implies that even an extended panel including *SPEN*, *SF3B1*, *FBXW7* and *MED12* mutations will neither be sufficient to identify all patients with abnormally strong NOTCH1 signalling. Approaches beyond the genomic level to measure NOTCH1 signalling strength are the detection of NICD1 at protein level or the assessment of a “NOTCH1 gene expression signature” as compiled by Fabbri et al. *via* an integrated analysis of gene expression profiling and NOTCH1 ChIP-Seq results (31). Importantly, the assessment of NOTCH1 signalling strength requires standardized conditions, as for example, CLL cells may not be collected in EDTA acting as strong inducer of NOTCH1 signalling due to its Ca^2+^-chelating ability (73).

Sufficiently large CLL and RT patient cohorts have hitherto not been screened systematically for the different mechanisms underlying NOTCH1 activation. The overall impact of NOTCH1 signalling on CLL progression is hence not appropriately addressed and it is yet unclear whether NOTCH1 affecting alterations such as *FBXW7* and *SPEN* mutations have the same effects on prognosis and treatment response than *NOTCH1* mutations. Due to the relatively low frequency of these mutations even in high-risk CLL cohorts, it will be challenging to seek clarity on this question. Targeted next-generation sequencing approaches assessing all recurrent mutations associated with NOTCH1 de-regulation will have to be applied to large-scale patient cohorts and likewise, more effort has to be invested to unravel causes for de-regulated NOTCH1 signalling beyond the genomic level.

## Author contributions

JE conceived and wrote the manuscript and created the figures. The author confirms being the sole contributor of this work and has approved it for publication.

## Funding

The article was supported in part by Alb Fils Kliniken, Göppingen, Germany.

## Conflict of interest

The author declares that the research was conducted in the absence of any commercial or financial relationships that could be construed as a potential conflict of interest.

## Publisher’s note

All claims expressed in this article are solely those of the authors and do not necessarily represent those of their affiliated organizations, or those of the publisher, the editors and the reviewers. Any product that may be evaluated in this article, or claim that may be made by its manufacturer, is not guaranteed or endorsed by the publisher.
